# Characterization of the institutionalization of pharmaceutical services in Brazilian primary health care

**DOI:** 10.11606/S1518-8787.2017051007135

**Published:** 2017-09-22

**Authors:** Gisélia Santana Souza, Ediná Alves Costa, Rafael Damasceno de Barros, Marcelo Tavares Pereira, Joslene Lacerda Barreto, Augusto Afonso Guerra, Francisco de Assis Acurcio, Ione Aquemi Guibu, Juliana Álvares, Karen Sarmento Costa, Margô Gomes de Oliveira Karnikowski, Orlando Mario Soeiro, Silvana Nair Leite

**Affiliations:** I Faculdade de Farmácia. Universidade Federal da Bahia. Salvador, BA, Brasil; II Instituto de Saúde Coletiva. Universidade Federal da Bahia. Salvador, BA, Brasil; III Departamento de Saúde. Universidade do Estado da Bahia. Salvador, BA, Brasil; IV Departamento de Farmácia Social. Faculdade de Farmácia. Universidade Federal de Minas Gerais. Belo Horizonte, MG, Brasil; V Departamento de Saúde Coletiva. Faculdade de Ciências Médicas da Santa Casa de São Paulo. São Paulo, SP, Brasil; VI Núcleo de Estudos de Políticas Públicas. Universidade Estadual de Campinas. Campinas, SP, Brasil; VII Programa de Pós-Graduação em Saúde Coletiva. Departamento de Saúde Coletiva. Faculdade de Ciências Médicas. Universidade Estadual de Campinas. Campinas, SP, Brasil; VIII Programa de Pós-Graduação em Epidemiologia. Faculdade de Medicina. Universidade Federal do Rio Grande do Sul. Porto Alegre, RS, Brasil; IX Faculdade de Ceilândia. Universidade de Brasília. Brasília, DF, Brasil; X Faculdade de Ciências Farmacêuticas. Pontifícia Universidade Católica de Campinas. Campinas, SP, Brasil; XI Departamento de Ciências Farmacêuticas. Universidade Federal de Santa Catarina. Florianópolis, SC, Brasil

**Keywords:** Health Manager, Pharmaceutical Services, organization & administration, Primary Health Care, Health Services Research, Unified Health System, Gestor de Saúde, Assistência Farmacêutica, organização & administração, Atenção Primária à Saúde, Pesquisa sobre Serviços de Saúde, Sistema Único de Saúde

## Abstract

**OBJECTIVE:**

To characterize the current stage of the institutionalization of pharmaceutical services in Brazilian cities.

**METHODS:**

This study is part of the *Pesquisa Nacional sobre Acesso, Utilização e Promoção do Uso Racional de Medicamentos* (PNAUM – National Survey on Access, Use and Promotion of Rational Use of Medicines), a cross-sectional, exploratory, and evaluative study composed by an information survey in a representative sample of cities, stratified by Brazilian regions. We interviewed municipal secretaries of health, responsible for pharmaceutical services, and pharmacists responsible for the dispensing of medicines. The variables selected from the interviews were grouped into five dimensions that defined three stages of pharmaceutical services institutionalization: incipient (0%-34.0%), partial (35.0%-69.0%), and advanced (70.0%-100%), estimated based on the interviewees’ answers. Frequencies were estimated with 95% confidence intervals. For the statistical association analysis, the Chi-square test was applied, with significance level of p<0.05.

**RESULTS:**

Our results show a partial and heterogeneous process of institutionalization of pharmaceutical services in Brazil, and an advanced stage in formal structures, such as the municipal health plans and the existence of a standardized list of medicines. The analysed variables in the “organization, structure, and financing” dimension configured stages that range from partial to advanced. The management presented partial institutionalization, positively showing the existence of computerized system, but also disparate results regarding the autonomy in the management of financial resources. Indispensable items related to the structure expressed disparities between the regions, with statistically significant differences.

**CONCLUSION:**

The study showed a partial and heterogeneous process of institutionalization of pharmaceutical services in Brazilian cities, showing regional disparities. Variables related to the normative aspects of institutionalization were positively highlighted in all dimensions; however, it is necessary to conduct new studies to evaluate the institutionalization of pharmaceutical services’ finalistic activities.

## RESUMO

**OBJETIVO:**

Caracterizar o estágio atual da institucionalização da assistência farmacêutica nos municípios brasileiros.

**MÉTODOS:**

Artigo integrante da Pesquisa Nacional sobre Acesso, Utilização e Promoção do Uso Racional de Medicamentos, um estudo transversal, exploratório, de natureza avaliativa, composto por um levantamento de informações numa amostra representativa de municípios estratificada pelas regiões do Brasil. Foram entrevistados os secretários municipais de saúde, responsáveis pela assistência farmacêutica e farmacêuticos responsáveis pela dispensação dos medicamentos. As variáveis selecionadas das entrevistas foram agrupadas em cinco dimensões, definindo-se três estágios de institucionalização da assistência farmacêutica: incipiente (0–34%), parcial (35%–69%), avançada (70%–100%), estimados com base nas respostas dos entrevistados. Foram estimadas frequências com intervalos de 95% de confiança. Para análise de associação estatística aplicou-se o teste do qui-quadrado, com nível de significância de p < 0,05.

**RESULTADOS:**

Os resultados mostram um processo heterogêneo e parcial da institucionalização da assistência farmacêutica no Brasil e grau avançado nas estruturas formais, como nos planos municipais de saúde e existência de lista padronizada de medicamentos. As variáveis analisadas na dimensão organização, estrutura e financiamento configuraram estágios que variam de parcial a avançado. A gestão apresentou grau parcial de institucionalização, revelando positivamente a existência de sistema informatizado, e resultados discrepantes no tocante à autonomia na gestão dos recursos financeiros. Itens imprescindíveis relacionados à estrutura expressaram disparidades entre as regiões, com diferenças estatísticas significantes.

**CONCLUSÕES:**

O estudo mostrou um processo de institucionalização heterogêneo e parcial da assistência farmacêutica em municípios brasileiros, revelando desigualdades regionais. Destacaram-se positivamente as variáveis relacionadas aos aspectos normativos da institucionalização em todas as dimensões, entretanto faz-se necessário novos estudos para avaliar a institucionalização das atividades finalísticas da assistência farmacêutica.

## INTRODUCTION

The trajectory of pharmaceutical services in Brazil points to advances and challenges to its consolidation as a State policy, which aims to provide comprehensive health care for the population. Evidence in the political, administrative, and social scopes indicate a gradual movement of institutionalization of pharmaceutical services (PS), congruous with the construction process of the Brazilian Unified Health System (SUS), which aims to guarantee the access to medicines and their rational use^25^ ^,^ ^26^.

The institutionalization of pharmaceutical services has been induced by the Brazilian Ministry of Health in recent decades. Normative and political actions affected the financing, structuring, and organization of PS in several operational areas of SUS. Several initiatives compose this process: the publishing of the National Drug Policy^19^, the 1^st^ National Conference of Medicines and Pharmaceutical Services^23^, the passing of the National Policy of Pharmaceutical Services^22^, and the mandatory adoption of the *Relação Nacional de Medicamentos Essenciais* (Rename – National List of Essential Medicines), which is updated every two years, as a parameter to access to medicines.

The founding of the Department for Pharmaceutical Services by the Brazilian Ministry of Health, in 2003, was an important step in the institutionalization of PS, by endowing them with proper structures to conduct this policy^32^. The financing – essential element to support of the policy of access to medicines –, also leads to the institutionalization of PS on SUS. The specific financing ordinances for PS ensure resources from the three management levels for the acquisition of medicines and the structuring of PS in the cities. The *Programa de Qualificação da Assistência Farmacêutica* (Qualifar: SUS – Pharmaceutical Services Qualification Program)^24^ stands out in this process, also providing professional training and qualification for individuals.

The process of institutionalization requires evaluation, because formal structures incorporated in the level of organizations would tend to support and provide stability to a set of actions, for instance, those performed by PS in organizations that compose SUS, in its several management and health care levels. This study, thus, corroborates Oliver’s definition of institutionalized actions as the ones that tend to be enduring, socially accepted, resistant to change and not directly dependent on rewards or monitoring of their permanence^27^.

In a study that aims to develop a proposal for monitoring and normative evaluation of basic PS, in the state of Mato Grosso, Freitas^13^ considered the institutionalization of PS as a transversal component that covers its logistical cycle and extends itself to the actions that guarantee the sustainability of the area, such as the formalization in the organization chart of the Municipal Secretariat of Health, the legalization of the municipal pharmaceutical network, and the participation of deliberative instances of health.

By defending the institutionalization of health evaluation, Contrandiopoulos^8^ states that the institutionalization of this practice will only take place if the evaluation is incorporated to the health care organizations’ routine, so as to integrate the planning and management process of policies and programs.

Studies regarding the institutionalization of PS are scarce. An experience report regarding the medicine selection in public hospitals in the state of Sergipe highlighted the importance of the theoretical/methodological framework of the Strategic Situational Management to implement and consolidate the *Comissões de Farmácia e Terapêutica* (PTC – Pharmacy and Therapeutics Committees), an essential tool in the institutionalization of the medicine selection process^32^.

Pharmaceutical services, as a transversal component in SUS – with the medicine as a strategic input –, induces other sectoral policies, especially those of scientific and technologic development^20^. Their institutionalization, in the course of the decentralization of health actions, proved itself a necessary process to improve the health system performance as a whole in the search for the universality and integrality of primary health care^18^.

The institutionalization processes have some common elements characterizing them, whether in the institutionalization trajectory of policies, programs, or of specific practices within the organizations. With rules and the adoption of formal structures of policies, programs, and practices, standardizations lead to the internalization of values, habits, and cultures that are incorporated to the organizations routine^2^ ^,^ ^34^.

The problematization of the institutionalization process of PS requires the consideration of its transversal and systemic nature, considering the internalization of procedures and routines in the organizations that compose the several operational areas of SUS. It encompasses a group of planning and managing activities of PS and, also, of practices that require management and technical expertise that entail the selection, scheduling, acquisition, and dispensing of medicines and of therapeutic planning activities of medical nature, aiming the rational use of medicines.

Such studies contribute to confirm the performance of governmental actions in the implementation and sustainability of PS policies. This study aimed to characterize the current stage of the PS institutionalization in the municipal health care systems in Brazil, seeking to contribute with the discussion regarding the implementation of PS policies.

## METHODS

This article is part of the *Pesquisa Nacional sobre Acesso, Utilização e Promoção do Uso Racional de Medicamentos – Serviços, 2015* (PNAUM – National Survey on Access, Use and Promotion of Rational Use of Medicines –Services, 2015), which aims to characterize the organization of PS in SUS primary health care, aiming to promote the access and rational use of medicines, as well as to identify and discuss the factors that interfere in the consolidation of PS in the cities.

PNAUM is a cross-sectional, exploratory, and evaluative study composed by an information survey in a sample of primary health care services of cities that represent the Brazilian regions, with direct observation of PS and interviews, and with a planned sample of 600 cities, carried out between 2014 and 2015. From these 600 cities, 300 were selected for an enquiry about health services, with face-to-face interviews with users, physicians, those responsible for dispensing of medicines, and direct observation of structural aspects.

We selected 27 capitals and 0.5% of the largest cities of each region, and we chose the remaining cities by lot. The estimation of the size of the representative sample considered three levels: cities, medicine dispensing services, and users. The sampling process aimed to maximize the randomization. The interviews were conducted by trained professionals, with the application of a structured questionnaire for each group of interviewees: face-to-face interview with physicians, those responsible for dispensing of medicines, and users; and phone interview with the professionals responsible for pharmaceutical services (RPS) and the municipal secretaries of health (SMS). More details can be found in the article by Álvares et al.^1^

This article used data from the interviews with SMS, those RPS, and those responsible for dispensing of medicines; in this scenario, we only used the data of pharmacists, since the analysed variable could not be applied to the other professionals. Regarding the SMS, the data were used only for Brazil because the percentage of interviewees did not represent each region. Concerning the RPS, the percentage of interviewees was representative, allowing the data to be used to characterize the institutionalization in the five regions as well as in Brazil.

The development of an operational concept was required for the institutionalization of PS in the cities, to express the necessary dimensions to the characterization of its stage.

For the analysis, we defined the institutionalization of PS in the cities as a social, political, and technical/administrative process that expresses itself in the creation of formal structures in the municipal health care systems, in the organization, structuring, and financing of PS, in management tools, in the development of practices and activities related to PS, in social participation and control, aiming to promote a comprehensive health care.

The operational elements were organized into five dimensions, with the selected variables, aiming to characterize the PS institutionalization in the cities (Table 1).

**Table 1:** Dimensions and variables selected for the characterization of the pharmaceutical services institutionalization in the cities. National Survey on Access, Use and Promotion of Rational Use of Medicines – Services, 2015.

Dimension of PS	Selected Variables
Formal structures	The coordination of PS is present in the organization chart of the Municipal Secretariat of Health
	PS are present in the Municipal Health Plan
	Existence of Comissões de Farmácia e Terapêutica (PTC – Pharmacy and Therapeutics Committees)
	Existence of standardized list of medicines
	Existence of Comissão Permanente de Licitação (CPL – Permanent Bidding Committee)
Organization, structure, and financing	Expenditure with the structuring of municipal PS
	Total application of the funds for PS
	The professional responsible for PS is a pharmacist
Management tools	The coordination of PS has total or partial autonomy in the management of financial resources
	Existence of SOP for reception, storage, dispensing, and delivery of medicines
	Existence of computerized system for PS management
	Provision of information on the medicine delivery locations to the population
	Existence of training for PS professionals
Practices and activities pertaining to PS	Performance of clinical activities
Social participation and control	Existence of mechanisms to receive criticism and suggestions from workers
	Existence of mechanisms to receive criticism and suggestion from users
	Municipal Health Council (CMS) discusses and deliberates about PS
	Existence of accountability of PS in the Municipal Health Council (CMS)
	Users participate in the decisions related to the PS management

PS: pharmaceutical services; SOP: standard operating procedures

In the data analysis for the characterization of the PS institutionalization, concerning the PTC, we only considered answers that confirmed the existence of this structure, and references to implementation processes were disregarded. Concerning the autonomy of the PS coordination in the management of financial resources (management tool), the answers of total or partial autonomy were considered as “yes.” In the variable *Conselho Municipal de Saúde* (CMS – Municipal Health Council), which discusses and deliberates over pharmaceutical services, the answers “always” and “repeatedly” were considered affirmatives.

Based on the percentage obtained from the means positive answer for each variable, we defined three stages of the PS institutionalization: incipient (0-34.0%), partial (35.0%-69.0%), and advanced (70.0%-100%). The data were analysed with the SPSS^®^ software, version 21, in the analysis module for complex samples. For the statistical association analysis, we applied the Chi-square test, with significance level of p<0.05.

PNAUM was approved by the National Research Ethics Committee (Opinion 398.131/2013). The objectives of the research were explained to the interviewees, and they signed the informed consent form.

## RESULTS

We interviewed 369 SMS (61.5% of the estimated sample); 507 RPS (84.5% of the estimated sample); and 1,139 professionals responsible for dispensing of medicines (83.6% of the estimated sample). 32.7% of the aforementioned were pharmacists.

Table 2 presents the characteristics of PS institutionalization in Brazil, according to the interviews with SMS and those RPS. Table 3 shows the characteristics of PS institutionalization in Brazil and its regions, according to those RPS. In general, the Southeast region showed more positive results and the North, more negative results.

**Table 2:** Pharmaceutical services institutionalization in Brazil*, according to those responsible for pharmaceutical services (n = 507) and municipal secretary of health (n = 369). National Survey on Access, Use and Promotion of Rational Use of Medicines – Services, 2015.

Dimension/variable	RPS% (95%CI)	SMS% (95%CI)
Formal structures		
The coordination of PS is present in the organization chart	82.7 (78.3–86.4)	91.7 (87.7–94.5)
PS are present in the Municipal Health Plan	92.3 (89.1–94.6)	97.2 (94.3–98.7)
Existence of Comissão de Farmácia e Terapêutica (PTC – Pharmacy and Therapeutic Committee)	13.2 (10.1–17.1)	10.7 (7.4–15.2)
Existence of standardized list of medicines	85.9 (82.1–89.0)	85.5 (80.9–89.1)
Existence of exclusive CPL for medicine acquisition	37.7 (32.6–43.0)	40.0 (34.0–46.3)
Organization, structure, and financing		
Expenditure with PS structuring	54.8 (49.1–60.5)	67.3 (61.1–73.0)
Total application of funds	86.4 (81.1–90.4)	97.1 (94.1–98.6)
Management tools		
The coordination of PS has total or partial autonomy in the management of financial resources	57.9 (48.7–68.0)	68.3 (62.3–73.8)
Existence of computerized system for PS management	70.8 (66.0–75.1)	74.7 (69.2–79.5)
Existence of training for PS professionals	11.9 (8.6–16.2)	37.7 (31.8–44.0)
Social participation and control		
Existence of mechanisms to receive criticism and suggestions from workers about PS	32.8 (27.8–38.3)	63.0 (56.8–68.8)
Existence of mechanisms to receive criticism and suggestions from users about PS	40.7 (35.2–46.4)	68.2 (62.2–73.7)
Municipal Health Council (CMS) discusses and deliberates always or repeatedly about PS	42.3 (35.9–48.8)	53.7 (47.3–59.9)
Existence of accountability of PS in the CMS	82.8 (76.4–87.7)	93.5 (89.4–96.1)

RPS: those responsible for pharmaceutical services; SMS: municipal secretary of health; PS: pharmaceutical services; CPL: Permanent Bidding Committee; CMS: Municipal Health Council.

^*^Institutionalization stages: incipient – 0 to 34.0%; partial – 35.0% to 69.0%; advanced – 70.0% to 100.0%

Source: PNAUM – Services, 2015.

**Table 3:** Pharmaceutical services institutionalizationa in Brazil, according to those responsible for the pharmaceutical services (n = 507). National Survey on Access, Use and Promotion of Rational Use of Medicines – Services, 2015.

Dimension/variable	Regions					

	North	Northeast	Midwest	Southeast	South	Brazil
					
	% (IC95%)	% (IC95%)	% (IC95%)	% (IC95%)	% (IC95%)	% (IC95%)
Formal structures						
The coordination of PS is present in the organizational chart	71.8 (61.0–80.6)	82.5 (72.2–89.6)	85.8 (76.5–91.8)	87.3 (78.5–92.8)	79.2 (69.6–86.3)	82.7 (78.3–86.4)
PS is present in the Municipal Health Plan^b^	88.0 (78.4–93.7)	100.0 (100.0–100.0)	91.3 (82.7–95.8)	88.8 (79.8–94.1)	88.3 (80.2–93.4)	92.3 (89.1–94.6)
Existence of Pharmacy and Therapeutic Committee	11.4 (6.4–19.5)	10.2 (5.1–19.1)	14.7 (8.9–23.5)	14.1 (8.3–22.8)	15.9 (10.0–24.3)	13.2 (10.1–17.1)
Existence of standardized list of medicines^b^	70.6 (60.3–79.1)	92.0 (83.2–96.4)	83.7 (74.7–90.0)	86.9 (78.3–92.4)	82.9 (74.1–89.1)	85.9 (82.1–89.0)
Existence of exclusive CPL for medicine acquisition	41.4 (31.2–52.4)	33.6 (23.5–45.6)	49.0 (38.5–59.5)	38.1 (28.5–48.7)	36.9 (27.8–46.9)	37.7 (32.6–43.0)
Organization, structure, and financing						
Expenditure with PS structuring	44.0 (32.9–55.8)	62.5 (50.5–73.2)	58.5 (47.0–69.1)	44.3 (33.0–56.2)	59.1 (48.4–69.0)	54.8 (49.1–60.5)
Total application of funds	73.7 (59.8–84.1)	80.5 (67.0–89.4)	85.3 (70.8–93.3)	90.2 (79.8–95.5)	92.7 (83.0–97.0)	86.4 (81.1–90.4)
The professional responsible for PS is a pharmacist^b^	86.6 (77.7–92.2)	83.7 (73.8–90.3)	88.1 (80.0–93.2)	94.8 (88.0–97.8)	95.0 (88.6–97.9)	90.3 (86.8–93.0)
Management tools						
The coordination of PS has total or partial autonomy in the management of financial resources	35.8 (26.1–46.8)	53.7 (42.3–64.6)	50.4 (40.1–60.7)	62.6 (51.8–72.3)	66.6 (56.6–75.3)	57.9 (52.4–63.1)
Existence of computerized system for PS management^b^	40.1 (30.5–50.5)	61.2 (50.0–71.4)	53.5 (43.5–63.3)	78.7 (69.2–85.8)	88.8 (80.9–93.7)	70.8 (66.0–75.1)
Existence of SOP for medicine reception^b^	62.0 (51.0–71.9)	68.7 (57.0–78.4)	66.4 (55.8–75.6)	78.8 (69.0–86.1)	58.7 (48.7–68.1)	68.9 (63.8–73.5)
Existence of SOP for storage^b^	60.8 (49.7–70.8)	72.0 (60.5–81.1)	70.2 (59.8–78.9)	80.5 (71.1–87.4)	60.9 (50.8–70.1)	71.2 (66.2–75.6)
Existence of SOP for dispensing^b^	53.3 (42.4–63.8)	67.2 (55.5–77.2)	68.8 (58.3–77.6)	77.2 (67.6–84.7)	56.8 (46.7–66.3)	67.1 (62.0–71.9)
Existence of SOP for delivery^b^	55.0 (44.1–65.5)	67.1 (55.4–77.0)	71.9 (61.5–80.4)	83.4 (74.2–89.7)	57.7 (47.7–67.1)	69.6 (64.6–74.1)
Existence of training for PS professionals	10.4 (5.3–19.2)	11.6 (5.6–22.4)	14.8 (8.4–24.9)	13.5 (7.6–22.8)	9.5 (4.8–17.8)	11.9 (8.6–16.2)
Those RPS participate in planning health actions of different technical areas	34.5 (24.8–45.7)	24.9 (15.1–38.0)	30.9 (21.2–42.5)	22.5 (14.7–33.0)	31.5 (22.2–42.4)	26.9 (22.1–32.3)
Provision of information on the standardized medicine delivery locations to the population	50.6 (39.9–61.2)	43.4 (31.5–56.0)	42.1 (31.4–53.6)	35.7 (26.1–46.7)	39.5 (29.5–50.4)	40.5 (35.0–46.2)
Social participation and control						
Existence of mechanisms to receive criticism and suggestions from workers about PS	29.2 (20.3–40.0)	24.2 (15.0–36.8)	42.2 (31.6–53.6)	35.9 (26.2–46.9)	36.5 (26.9–47.3)	32.8 (27.8–38.3)
Existence of mechanisms to receive criticism and suggestions from users abour PS	43.6 (33.1–54.7)	32.5 (21.9–45.3)	42.2 (31.6–53.6)	43.1 (32.6–54.2)	45.6 (35.2–56.4)	40.7 (35.2–46.4)
Municipal Health Council discusses and deliberates always or repeatedly about PS	34.0 (23.2–46.8)	30.2 (18.1–45.8)	39.0 (27.6–51.8)	31.7 (21.4–44.1)	36.8 (25.9–49.2)	33.4 (27.5–39.8)
Accountability of PS in the CMS	81.4 (66.9–90.5)	89.4 (73.2–96.3)	83.8 (70.5–91.8)	77.5 (63.7–87.1)	83.8 (70.8–91.6)	82.8 (76.4–87.7)

CPL: Permanent Bidding Committee; PS: pharmaceutical services; SOP: standard operating procedures; CMS: Municipal Health Council

^a^ Institutionalization stages: incipient – 0 to 34.0%; partial – 35.0% to 69.0%; advanced – 70.0% to 100.0%

^b^ p < 0,05

Source: PNAUM – Services, 2015.

In the “formal structures” dimension (Table 2), of the five variables analysed, three had means above 80.0%: PS in the Municipal Health Plans (PMS); existence of a standardized list of medicines; and PS coordination in the organization chart of the municipal secretariats of health, with percentage differences between the informants.

Regarding the presence of PS in the PMS and the existence of a standardized list of medicines, the distribution in the regions presented high percentages and statistically significant differences (Table 3). In Brazil, PTC have a low percentage, according to those RPS (13.2%) and SMS (10.7%) (Table 2). This result contrasts with the high percentage of cities with standardized list of medicines, which may indicate the adoption of lists that are available on SUS, such as Rename from the Brazilian Ministry of Health.

Regarding the existence of *Comissão Permanente de Licitação* (CPL – Permanent Bidding Committee) exclusively for the acquisition of medicines, Brazil presented a percentage that ranges from 37.7% to 40.0%, according to those RPS and SMS, respectively (Table 2). In the Brazilian regions, the lowest percentage was found in the Northeast (Table 3).

In the organizational, structural, and financing dimensions, the analysed variables (Table 2) showed the stage of PS institutionalization is between partial and advanced. Table 3 highlights the high percentage of pharmacists responsible for PS in Brazilian regions, the Northeast with the lowest percentage (83.7%), and statistically significant differences. However, PNAUM points out that, in Brazil, a little more than 40.0% of pharmacies/medicine dispensing units has a pharmacy technician in charge^9^.

Regarding the financing of PS in Brazil, 86.4% of those RPS claimed they applied the complete value of the municipal financing funds; among SMS, the percentage was 97.1% (Table 2). These percentages varied between regions, without statistically significant differences (Table 3).

Regarding the financial resources expenditure AF structuring, 54.8% of the RAF stated that these expenditures occurred. For the SMS this percentage was 67.3% (Table 2). The North and South regions were below the national the mean (Table 3), with no statistically significant differences between the regions.

The “management tools” dimension presents a partial institutionalization stage in Brazil. In general, the region that most stood out was the Southeast, with advanced stages in five out of the nine analysed variables, contrasting with the North region (Table 3).

In Brazil, regarding the autonomy of the management of financing resources by the PS coordination, we found discrepancy between those RPS (57.9%) and SMS (68.3%) (Table 2). In this component, the regions presented great differences between themselves: 35.8% in the North and 66.6% in the South, without statistically significant difference.

Regarding the existence of a computerized system for pharmaceutical services management, the percentages in Brazil were above 70.0%, according to both interviewees (Table 2). However, we found great differences between the regions: 88.8% in the South and 40.1% in the North (Table 3), with statistically significant differences. Among the RPS who confirmed the existence of a computerized system in PS management, only 16.3% stated they use the Hórus System, made available and recommended by the Brazilian Ministry of Health.

The standard operating procedures (SOP) for the receiving, storage, dispensing, and delivery of medicines (Table 3) are above 67.0% in Brazil, with statistically significant differences between the regions, highlighting the Southeast in the advanced stage.

PNAUM showed a concerning situation about the qualification of the professionals working in PS in Brazil. Only 11.9% of those RPS affirm the existence of qualification/training for the professionals of pharmaceutical services, and this percentage rises to 37.7% of the SMS (Table 2).

Those RPS participated slightly in the planning process of the other technical areas of health, confirmed by 26.9% of these agents, without statistically significant difference (Table 3).

In Brazil, only 40.5% of those RPS provided information to the population about the locations of medicine delivery. The differences between the regions, however, presented no statistical significance (Table 3).

In the “social participation and control” dimension, PNAUM showed that, in three out of the four analysed variables, the institutionalization is incipient in Brazil, according to those RPS and partial, according to SMS (Table 2). The achieved percentages of the variable regarding the accountability of PS in the CMS were high both for those RPS (82.8%) and SMS (93.5%). However, the CMS discusses and deliberates always and repeatedly about PS for 42.3% of those RPS and 53.7% of SMS.

Concerning the permeability of PS for users, there is a great difference between the agents: 68.2% of SMS and 40.7% of those RPS affirm the existence of mechanisms to receive criticism and suggestions about PS. Regarding the mechanisms to receive criticism and suggestions from health professionals, 63.0% of SMS and 32.8% of those RPS answered positively (Table 2). The Midwest and the South achieved better results, without statistically significant differences between the regions (Table 3).

In the “PS practices” dimension, only pharmacists responsible for the medicine delivery answered the questions about the variable regarding the performance of clinical activities. The institutionalization stage was incipient because only 21.3% of pharmacists stated they perform these activities.

## DISCUSSION

Our study showed a heterogeneous scenario of the PS institutionalization in the Brazilian regions. The “formal structure” dimension reached the highest score. Portela et al.^31^ consider that the presence of PS in the organization chart of the SMS and the existence of their own organizational structure characterize their institutionalization. However, formal structure and regulations compose a dimension, and they are only the initial steps of a policy institutionalization process.

The research showed that the PS formal structures contain variables that indicate the institutionalization in the advanced stage and others in partial or incipient stages, such as the low rate of PTC.

The formalization of PS in the organization chart validates its operation before the public areas, making it visible to the population and the remaining health sectors^7^. The low rate of PTC corroborates the results of Marques & Zucchi^17^, which point to these committees’ structural weaknesses in Brazil, structures that are important to the selection, standardizing, and rational use of medicines and that should be adjusted to the national and local situations^17^. However, the difficulties of Brazilian cities in the health care system organization and structuring may hinder the structuring of these committees^16^.

The medicine selection and acquisition are part of the logistic cycle of PS. The selection must result in a standardized list that guides the acquisition, which, in turn, takes place in the medicine purchase process in suitable quantity and quality, aiming to lower the costs and the supply system regular operation^19^. The absence of an exclusive CPL may hinder the expenditure of financial resources destined to the medicine acquisition because of the possibility of this item’s lack of priority compared with acquisitions from other municipal administrations.

The “PS organization, structure, and financing” dimension shows the most unfavourable percentages in the variable concerning the expenses with the structuring of services. The lack of proper structuring of PS may negatively affect the working conditions and medicine sanitary condition, with possible losses and risks in their use^10^.

Despite the difference between agents regarding the use of PS funds, these results stand close to a research about Paraiba cities^6^. In that study, after ten years of the enactment of PNAF, the investment in the structuring of Brazilian public pharmacies was still low regarding medicine storage conditions^35^ ^,^ and its results corroborated the research of the Pan American Health Organization^21^, in which only 61.0% of the pharmacies had suitable conditions for the medicine preservation in warehouses and 60.0% in the municipal pharmaceutical supply centers^28^.

The tripartite financing for medicine acquisition has been important to the decentralization of PS, and it has grown since the establishment of specific financing for its structuring^21^. However, the increase in financing in the last years was insufficient to qualify the medicine acquisition by the public agents in Brazil, which is a challenge for the PS management to improve expenditure in the area. The lack of autonomy of the coordination of PS regarding the financial resources, which those RPS and SMS show with significant percentages, may explain part of the difficulties regarding the pharmaceutical services structuring and qualification.

Although in Brazil the percentages of those who claim having a computerized system are above 70.0%, we found great differences between the regions, with inequalities in the infrastructure and logistics of the health services. The low application of *Sistema Hórus* (Hórus System) attests to this, with evidences that this system strengthens the control and the monitoring of medicine use^11^.

The high percentages of those RPS that confirm the existence of several SOP stand out. They are important elements to the organization and quality of work, because they describe the sequence of every crucial step that should be taken to ensure the expected result of the task^5^. Similarly, medical protocols and therapeutic guidelines are tools that contribute to the adequate use of medicines^33^.

The presence of qualified professionals is fundamental; PS are a multidisciplinary and dynamic process, with actions that aim to provide access, quality, and rational use of medicines^14^. Professional training, mainly that of the pharmacist, is necessary to the structuring of PS processes, from the technical to the administrative issues, related to the logistic cycle and pharmaceutical care^14^.

PNAUM showed a management process of PS that is still restricted to the sector, which may compromise its transversality in the support to health care networks and their administration^18^. The integration of professions is indispensable to the development of health practices in a comprehensive approach^29^. Teamwork should be in the frontline of strategies to change the health care models, before the extremely complex sociocultural and economic contexts that are increasingly more dynamic^30^.

The “social participation and control” dimension has a significant role in the institutionalization process of public policies, especially in the State actions and public resources destination, aiming to resist the reduction of social policies, the privatization, and the commodification of the services^9^. Health Councils have become the most comprehensive networks of participatory instances of the country^12^. The social control instances are not mechanisms above society and immune to conflict of interest and disputes focused on distinct societal projects^4^. PNAUM shows that social control is still limited in PS, despite of conflict of interest and of the symbolic and economic power that permeates medicines, which are strategic elements in this field of health.

Access to medicines is in the population’s interest, thus they have increasingly sought the judicial system to ensure this right^3^ ^,^ ^6^ ^,^ ^15^.However, PNAUM shows that CMS are instances insufficiently sought for the right to PS. The process of social participation and control show weaknesses regarding PS in Brazil. Nevertheless, the high percentages for the variable of accountability of PS in the CMS stand out, which leads us to believe that legal obligations affect this type of result.

In all variables of the institutionalization of PS, the answers of SMS presented higher percentages compared with the answers of those RPS, except for the existence of PTC. SMS may express a more optimistic perspective because they have the role of administrators of the municipal health policies. However, it was not possible to deepen the analysis of these differences, which is one of the study’s limitations as an exploratory research. This constitutes an outline for future studies.

Our results allowed us to conclude that the PS institutionalization process in the municipal health care systems is still partial and heterogeneous in the different regions of Brazil. We observed more advances in formal structures, since they are necessary conditions to the beginning of the institutionalization process of PS policies.

In general, variables related to normative aspects scored higher, except in finalistic activities, in the integration with other technical areas, and in the social participation and control. The complete institutionalization of PS as a systemic and permanent process presupposes that the finalistic activities of PS are disseminated in the health services.

The organization, structure, and financing variables reached a high score. These are conditioning results to the institutionalization and sustainability of PS policies on SUS, which relies on a model of practices consistent with the comprehensiveness of health care. The consolidation of a public policy takes place when society recognizes it as a right and permanent action of the State. However, according to findings related to social participation and control, the PS policies in Brazil have yet to reach this position.

We did not find researches concerning the PS institutionalization in municipal health care systems in Brazil to make comparisons, which was another limitation of this study. Nevertheless, we were able to point indicators and subsidies for further evaluations. With PNAUM, we concluded that PS institutionalization in the municipal scope is an ongoing process that already presents important advances, although several aspects are insufficiently consolidated to resist the restrictive political contingencies of the right to health.
